# Supplementary material for: IFT88 controls NuMA enrichment at k-fibers minus-ends to facilitate their re-anchoring into mitotic spindles
Source: Sci Rep. 2019 Jul 16;9:10311. doi: 10.1038/s41598-019-46605-x (PMC6635507; doi:10.1038/s41598-019-46605-x)
Supplement: Supplementary file 1 — Supplementary Figure 1 to 3 [file 41598_2019_46605_MOESM1_ESM.pdf]

IFT88 controls NuMA enrichment at k-fibers minus-ends to facilitate their re-anchoring into mitotic spindles

Nicolas TAULET[1*✉], Audrey DOUANIER[1*], Benjamin VITRE[1], Christelle ANGUILLE[1], Justine MAURIN[1], Yann DROMARD[2], Virginie GEORGET[2] and Benedicte DELAVAL[1✉].

[1] CRBM, CNRS, Univ. Montpellier
Centrosome, cilia and pathologies Lab
1919 Route de Mende, 34293 Montpellier, France

[2] Montpellier Ressources Imagerie
CRBM, CNRS, Univ. Montpellier
1919 Route de Mende, 34293 Montpellier, France

* NT and AD have contributed equally to the work

✉ Correspondence:
benedicte.delaval@crbm.cnrs.fr
nicolastaulet@hotmail.com

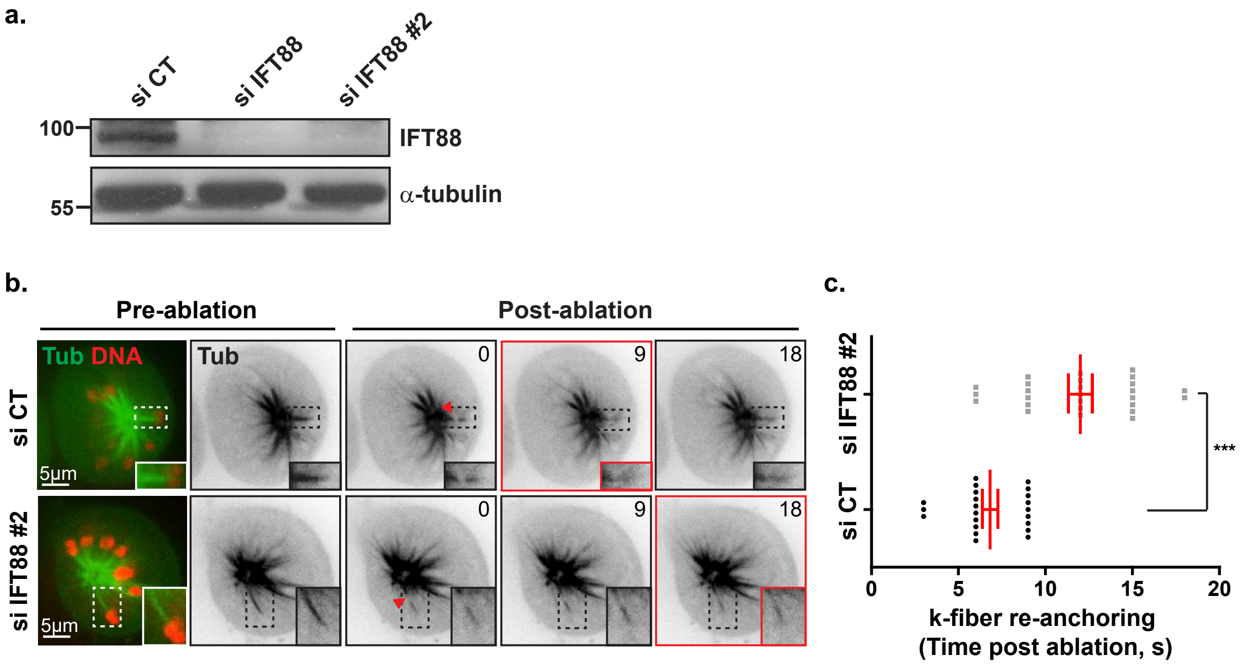

Supplementary Fig. 1. IFT88 knockdown affects k-fibers re-anchoring into spindle. (a) Western-blots showing the amount of IFT88 in GFP-α-tubulin LLC-PK1 cells transfected with control (CT) or two different IFT88 siRNA oligos (IFT88 and IFT88 #2). α-tubulin: loading control. **(b)** Images from time-lapse microscopy of monopolar GFP-α-tubulin LLC-PK1 labelled for DNA (Hoechst live, red), to allow for k-fibers detection, in control (CT) and IFT88-depleted cells (oligo IFT88 #2) (left panels). Inverted contrast images of α-tubulin before and after k-fiber ablation (ablation site, red arrowhead) show a delay in k-fiber re-anchoring into spindle upon IFT88 depletion. Time post-ablation (s). Single planes are shown. Insets: magnification of the ablated k-fibers, dashed boxes regions. Red boxes indicate k-fibers re-anchoring. **(c)** Quantification of the time (s) required for k-fiber re-anchoring into the main spindle after laser ablation in CT and IFT88-depleted cells (oligo IFT88 #2). n ≥22 ablated k-fibers (1 ablated k-fiber per cell), 2 experiments. Mean +/- s.e.m ***P < 0.001 compared to control (t test). Scale bars: 5 µm.

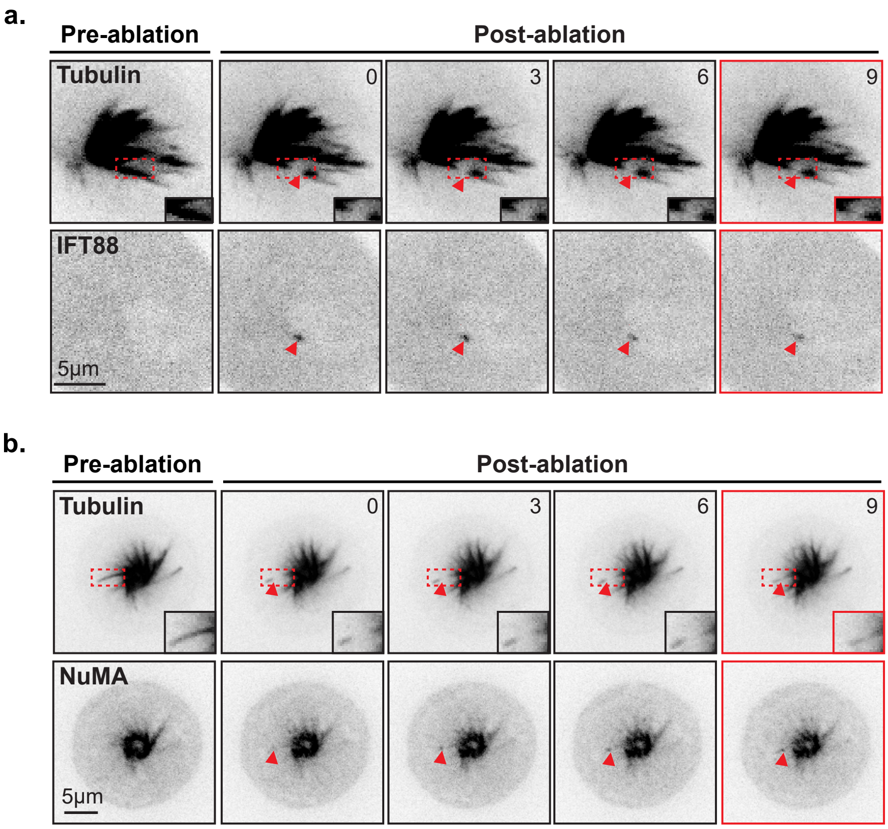

Supplementary Fig. 2. IFT88 and NuMA accumulation at k-fibers minus-end after laser ablation. Inverted contrast images from time-lapse microscopy of monopolar Emerald-IFT88 LLC-PK1 cells **(a)** and YFP-NuMA LLC-PK1 cells **(b)** labelled for tubulin (SiR-Tubulin) before and after k-fiber laser ablation. Hoechst live was used to identify k-fibers attached to chromosomes. The data show that IFT88 accumulates at the newly generated k-fiber minus-end before NuMA and then decreases as k-fibers re-anchor to the main spindle (see line scans for fluorescence intensity measurements in Fig. 2d). Time post-ablation (s). Maximal projections (a) or single planes (b) are shown. Insets: magnification of the ablated k-fibers, dashed boxes regions. Red boxes indicate k-fibers re-anchoring. Scale bars: 5 μm.

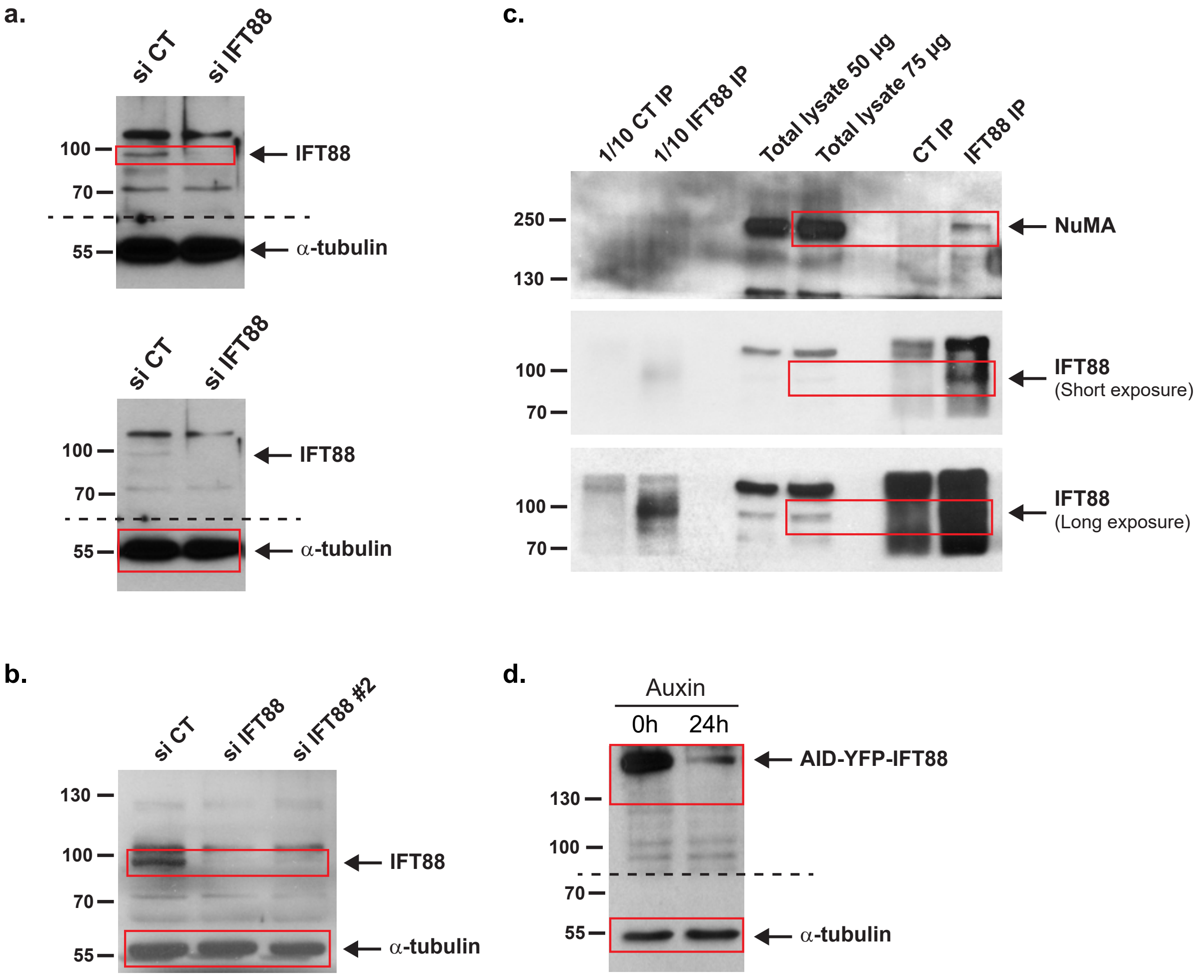

Supplementary Fig. 3. Uncropped images for presented western-blots. (a) Corresponds to Fig. 1c. Long (top) and short (bottom) exposures are shown. **(b)** Corresponds to supplementary Fig. 1a. **(c)** Corresponds to Fig. 2e. **(d)** Corresponds to Fig. 3e.
